# A Dog as an Out-Patient

**Published:** 1887-08-06

**Authors:** F. O. Morris

**Affiliations:** Rector of Nunburnholme, Yorkshire; author of "A History of British Birds," etc.


### A DOG AS AN OUT-PATIENT.

THE Porter (George Till) at King's College Hospital writes:—A curious case of canine sagacity came under my notice at this hospital this (Sunday) morning. While engaged in the hall I heard the bark of a dog, and on going out to drive it away I found to my surprise three dogs on the steps. Two of them ran off on seeing me, leaving the third one lying on the steps in a pool of blood, looking very exhausted. I patted it on the head and took hold of its leg, and found a cut three inches in length, and blood flowing freely from an artery. At the time one of our medical gentlemen came in, and I asked him if he would mind looking at it, which he did, and bathed the wound in lotion and bandaged it up, thus stopping the bleeding by pressure, and but for our timely aid the dog must have bled to death.

From what transpired, I traced the blood-stained paw-marks round the back of the hospital to Yates' Court, where apparently the dog had crawled through a hole in the boarding belonging to the Royal Courts of Justice, the dog no doubt having trodden on a piece of glass, which was there, and from the appearance of Yates' Court the poor animal must have lost a great quantity of blood. The singular feature of the story is that the animal hobbled unhesitatingly with his two canine friends direct round to the front steps of the hospital, where I found him as stated above, thus showing the sagacity of our canine friends.

(*To be continued.*)

